# Bacteriological Technology for Physicians

**Published:** 1891-01

**Authors:** 


BACTERIOLOGICAL TECHNOLOGY FOR PHYSICIANS, By DR. T. C. SALOMONSEN. Translated by William Trellase. Pp. 162; 8vo. New York: William Wood & Co. 1890.

The second edition of this well-known work has undergone some modifications and additions, greatly adding to its worth and completeness. In nearly all the chapters there has been a reëlaboration to keep it in the front of bacteriological investigations, which is making such rapid strides at the present day. For the inquiring physician this little work answers every purpose, and will enable him to read intelligently upon this important subject as well as make original researches. W. C. K.

